# Gender and equity considerations in AMR research: a systematic scoping review

**DOI:** 10.1007/s40592-024-00194-2

**Published:** 2024-04-27

**Authors:** Ingrid Lynch, Lorenza Fluks, Lenore Manderson, Nazeema Isaacs, Roshin Essop, Ravikanya Praphasawat, Lyn Middleton, Bhensri Naemiratch

**Affiliations:** 1 Human Sciences Research Council, Cape Town, South Africa; 2 University of the Witwatersrand, Johannesburg, South Africa; 3 Mahidol-Oxford Tropical Medicine Research Unit, Faculty of Tropical Medicine, Mahidol University, Bangkok, Thailand; 4 University of KwaZulu-Natal, Pietermaritzburg, South Africa

**Keywords:** AMR, Gender, Equity, Intersectionality, LMICs

## Abstract

Research on gender and antimicrobial resistance (AMR) beyond women’s biological susceptibility is limited. A gender and equity lens in AMR research is necessary to promote gender equality and support the effectiveness, uptake, and sustainability of real-world AMR solutions. We argue that it is an ethical and social justice imperative to include gender and related intersectional issues in AMR research and implementation. An intersectional exploration of the interplay between people’s diverse identities and experiences, including their gender, socio-economic status, race, disability, age, and sexuality, may help us understand how these factors reinforce AMR risk and vulnerability and ensure that interventions to reduce the risk of AMR do not impact unevenly. This paper reports on the findings of a systematic scoping review on the interlinkages between AMR, gender and other socio-behavioural characteristics to identify priority knowledge gaps in human and animal health in LMICs. The review focused on peer-reviewed and grey literature published between 2017 and 2022. Three overarching themes were gendered division of caregiving roles and responsibilities, gender power relations in decision-making, and interactions between gender norms and health-seeking behaviours. Research that fails to account for gender and its intersections with other lines of disadvantage, such as race, class and ability, risks being irrelevant and will have little impact on the continued and dangerous spread of AMR. We provide recommendations for integrating an intersectional gender lens in AMR research, policy and practice.

## Introduction

Antimicrobial resistance (AMR) poses a severe and immediate global threat to human and animal health, food security, and food safety (Medina-Pizzali et al. 2021). Elevated resistance levels significantly threaten the effectiveness of existing treatments for prevalent bacterial, viral, and fungal infections. If increasing resistance continues, it could reverse the progress in controlling infectious diseases and the medical advancements achieved during the 20th century (Jamrozik and Selgelid 2020). AMR’s effects, however, are spread disproportionately. Low- and Middle-Income Countries (LMICs) bear the brunt of the global AMR burden, with sub-Saharan Africa (SSA) reporting the highest death rate attributable to drug-resistant infections globally (Antimicrobial Resistance Collaborators 2022; Chereau et al. 2017; Elton et al. 2020; Varma et al. 2018). Social, structural and systemic challenges are critical drivers of the growing burden of AMR within LMICs. These challenges include “inappropriate prescription practices, inadequate patient education, limited diagnostic facilities, inappropriate (or unsuitable) sale of antimicrobials, [poorly] functioning drug regulatory mechanisms, and non-human use of antimicrobials such as in animal production” (Ayukekbong et al. 2017; p. 1).

The cost of AMR to the economy, including social and labour-loss effects, is significant and varies by country (Ahmed & Khan, 2020; Dadgostar 2019). The cost of AMR in Europe is estimated to be more than nine billion euros per annum. In the United States, AMR costs more than 20 billion dollars in direct healthcare expenses, excluding an estimated 35 billion dollars in productivity loss per annum (Dadgostar 2019). While the economic cost of AMR is felt globally, it is estimated that AMR would increase the poverty rate and significantly bear upon LMICs compared to the rest of the world (World Bank, cited in Dadgostar 2019). In addition to death and disability, prolonged illness results in extended hospital stays, the need for more expensive medicines when common first-line drugs prove ineffective, and financial challenges for those impacted (Ahmad & Khan, 2019; Dadgostar 2019; WHO 2021). This is especially problematic where individuals must cover the costs of medicines and hospital stays out of pocket, both in LMIC and among people in High-Income Countries (HICs) without adequate health insurance. Current evidence informing AMR mitigation is predominantly generated in resource-rich HICs (Ayukekbong et al. 2017; Wu et al. 2022). Curbing irrational antimicrobial use in human health forms a key focus in research, and the misuse and overuse of antimicrobials are seen to be the main drivers in the development of drug-resistant pathogens (WHO 2021).

However, as described elsewhere (Gautron et al. 2023; Tompson et al., 2021; Torres et al., 2021), and as we reiterate below, intervention and implementation research and the development of programs need to be contextually embedded to drive AMR solutions in LMICs. Pervasive poverty and inequality at individual and community levels provide the backdrop for and influence some of the key drivers of antibiotic use. Lack of clean water and sanitation and inadequate infection prevention and control promote the spread of microbes, some of which may already be resistant to antimicrobials, and others develop this. However, poor communities and ineffective municipal governments often lack the financial and technical resources to expand and maintain infrastructure. Consequently, individual behaviour is routinely identified as the most effective point of intervention despite the discordance between public health and community household understandings of medications and their uses. This is often seen as capable of redress through AMR education and awareness raising in human health and livestock-keeping settings. However, as Dixon and colleagues report: “Many of the antibiotic use practices that would be considered ‘irrational’ from a biomedical perspective (e.g., storing antibiotics for later, sharing them with others and using informal providers) are highly rational within the material and moral worlds inhabited by people living in contexts of scarcity and precarity” (2021, p. 2).

## The relationship between gender-based and other equity considerations and AMR

Gender equality is an international development priority and cuts across AMR mitigation in human, animal, and environmental health. Gender norms and practices are hidden drivers of persisting inequalities, shaping educational and economic opportunities, access to and control over resources, service utilisation, and decision-making power. As a result, women, men and other diverse genders are differently exposed to and impacted by AMR, and gender inequalities influence who can access, use and benefit from ways to tackle AMR (van der Heijden et al. 2019; WHO 2018). Attention to sex-disaggregated AMR and antibiotic use (AMU) data and analyses is growing and has yielded important insights. For instance, women are more prone to urinary tract infections (UTIs) due to their anatomy, poor sanitation, sexual violence, and other trauma (Minardi et al. 2011). This increases their lifetime antibiotic use compared to men and reduces the effectiveness of first-line antimicrobials in treating the infection (Medina and Castillo-Pino 2019). Pregnancy, childbirth, abortion and post-abortion care all increase women’s exposure to hospital-acquired infections (ReAct 2020).

Research on gender and AMR beyond women’s biological susceptibility is limited (ReAct 2020; WHO 2018). In their review of existing scholarship, Tompson and Chandler conclude that “little attention has been paid to gendered aspects of antibiotic use [and] research is needed to understand how to tailor stewardship initiatives better and reduce unintended harm in the face of these dynamics” (2021, p. 5). Limited research on AMR explores the interplay between people’s diverse identities and experiences, including their gender, socio-economic status, race, disability, age, and sexuality. Adopting an intersectional approach may help us understand how these factors reinforce AMR risk and vulnerability (Charani et al. 2021). Integrating an intersectional gender lens in AMR research will also ensure that interventions to reduce the risk of AMR do not impact unevenly (Manderson et al. 2009). Addressing AMR requires that gender-based and other equity considerations be considered to develop solutions that favour everyone.

As already noted, the burden of AMR is disproportionately spread globally and particularly pronounced in LMICs. The US Centres for Disease and Prevention (CDC) estimates that nearly fifty per cent of the world’s population lacks adequate sanitation, and one-third lack basic hygiene services (CDC, 2022). Poor water, hygiene and sanitation services increase the risk of infections, including water-borne and water-wash infections that might be managed through antibiotics. The lack of facilities also increases the likelihood of spreading resistance through the environment, including in soil and groundwater. Women and different marginalised populations are especially vulnerable because of the roles they fulfil in families, with heightened exposure to pathogens when caring for sick family members, and their economic roles, including managing domestic livestock, maintaining domestic gardens, and so on. Women present more often at primary health care with infections, although whether this reflects their overall great use of local clinics, including for the care of others, or because of the greater prevalence of infections as well as other health needs is unclear.

Our research builds upon Dixon colleagues’ (2021) exploration of the relationships between structural inequalities in LMICs and AMR. Guided by a review by Gautron and colleagues (2023) which outlines the interplay between biological (sex) and psychosocial (gender) constructs shaping individual’s health behaviours and experiences, we narrow our focus to consider research that directly addresses both AMR and gender (not sex). Our aim is to assess the extent to which AMR research in LMICs incorporate a gender lens, examining thematic focuses across such studies in both human and animal health.

## Methodology

The systematic scoping review aimed to synthesise existing evidence about the interconnections between AMR, gender (as a social construct), and socio-behavioural characteristics relevant to human and animal health in LMICs. The review entailed the following steps: (1) determine inclusion and exclusion criteria (e.g., time period, language of publication); (2) identify appropriate keywords and create Boolean phrases; (3) conduct the database search using the identified keywords; (4) screen the titles and abstracts of identified literature for relevance; (5) critically appraise the final dataset of included literature; (6) extract data from the full-text literature and synthesise; (7) and interpret and write up the findings (Snyder 2019; Tawfik et al. 2019). This methodology is rigorous, in-depth, and comprehensive.

### Search strategy

We used EBSCOhost, PubMed and ERIC– all aggregator databases that collate content from various publisher databases and provide access to peer-reviewed and grey literature– as well as Google Scholar. We applied the following inclusion and exclusion criteria:

- The search period was restricted to five years spanning 2017–2022, to synthesise current evidence and thereby provide recommendations that are based on up-to-date and relevant research.
- We included both peer-reviewed and grey literature such as reports and guiding documents produced by global bodies (e.g., WHO and United Nations institutions) and multinational- or regional bodies. This approach ensured a multi- and interdisciplinary focus, underpinned by a One Health approach, incorporating both human and animal health.
- Publications that made specific reference to LMICs were included, and consistent with this interest, the search was unrestricted in terms of the publication language, i.e., it was not limited to English language publications. The search did not, however, yield any relevant publications in languages other than English.
- In addition to peer-reviewed articles, the search included research reports, guidance documents, policy briefs, dissertations, conference papers, working papers and poster presentations. Documents excluded from the search were: books (and book chapters and book reviews), media (newspaper articles and blogs), commentaries, and conference abstracts and papers.

We used a combination of the following keywords for the search: gender, antibiotic use, antibiotic exposure, antimicrobial resistance (AMR), and antibiotic resistance. The database search was supplemented by backward citation tracing or backward snowballing, a search strategy where the reference lists of identified articles are reviewed for relevant articles not picked up in the database search (Hirt et al. 2021). This strategy is useful in scoping reviews where there is a small body of literature. It aids in identifying studies highly relevant to the review but not indexed using the study search terms. This often yields higher-quality results than systematic keyword searches.

**Fig. 1:**
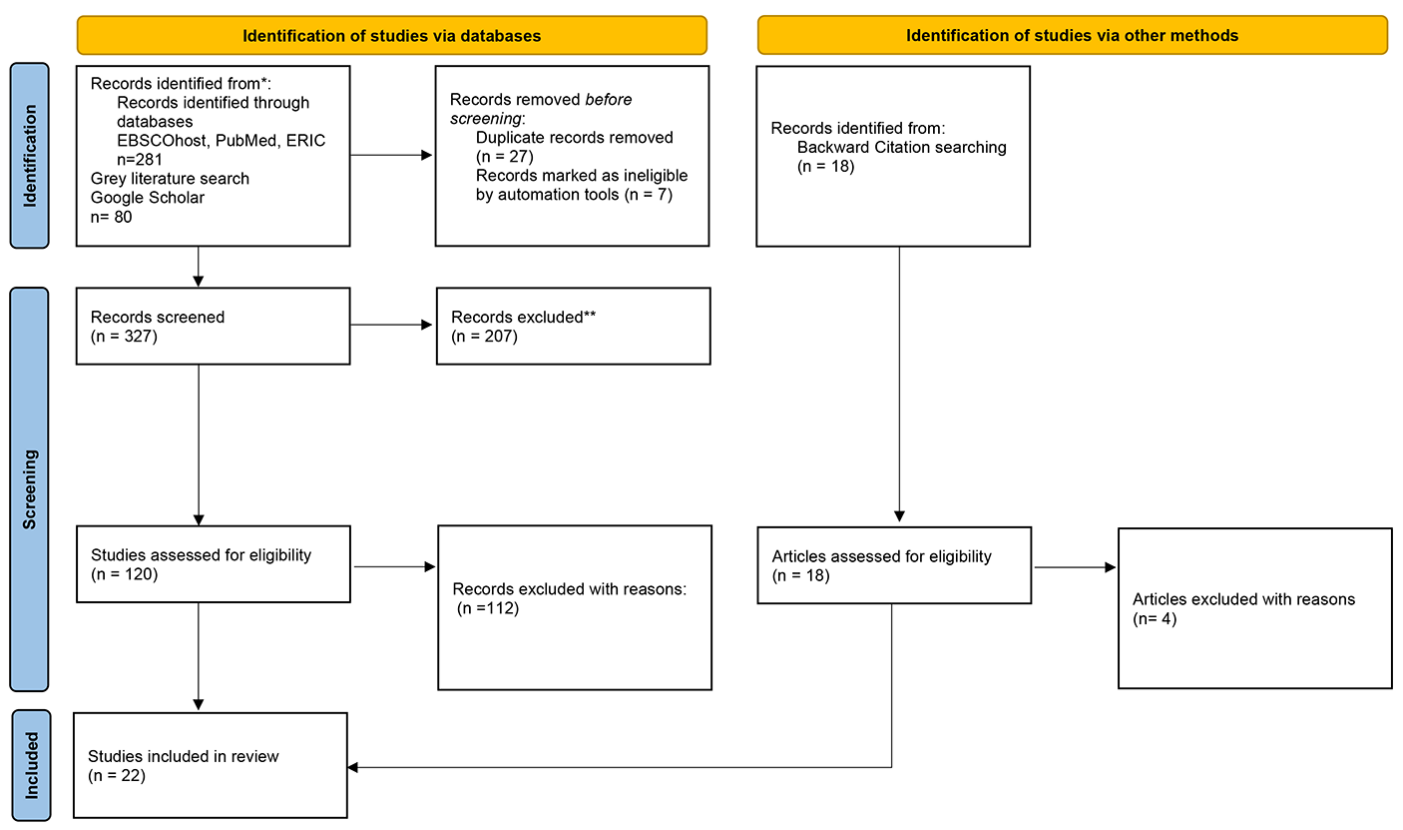
PRISMA Diagram

The titles and abstracts of the documents were screened for relevance against the inclusion and exclusion criteria by five project team members, adhering to a jointly developed scoping protocol and a screening guide. When relevance could not be ascertained from the abstract, the full paper was retrieved and further reviewed to determine relevance. In cases where the project team did not agree, all members reviewed the full paper to decide on its relevance to the project. The screening process yielded a final dataset of 22 articles for inclusion, with the steps outlined in Fig. 1.

### Coding and analysis

We developed a set of *a priori* codes, informed by the research objectives. These were further refined through an iterative process to capture evolving insights from the dataset. Where new codes were developed or existing codes refined, these were applied across all documents in the dataset. Once coded, the dataset was analysed using thematic analysis, aimed at “identify[ing] patterns and themes, done iteratively through referring to primary data sources where needed” (Lynch et al. 2017; p. 5). This entailed synthesising the coded data into potential themes, reviewing themes in relation to the dataset, generating a thematic map, and generating clear definitions and names for themes (Braun and Clarke 2012).

## Findings

Of the articles identified through the scoping review, the majority are focused on AMR research concerning human health (59%), followed by animal health (27%), and human and animal health (10%), with one article only that spans all One Health domains (human, animal and environmental health). Across all articles, gender is predominantly treated as binary, with only one article engaging sexual and gender diversity. Socio-behavioural factors intersecting with gender span a wide range, with class, education, age, and geography featuring most often. The remaining stratifiers include occupation, marital status, disability, sex, employment and sexuality. Research settings are also diverse, spanning household, community, healthcare, agricultural and educational settings. Table 1 provides a summary of the main characteristics of articles. Three overarching themes were identified in the dataset: (i) Gendered division of roles and responsibilities, (ii) gender power relations in decision-making and access to resources, and (iii) interactions between gender norms and health-seeking behaviours.

**Table 1:** Summary table of documents included in review

First author		First author location	Article type	Research design	Study country / region	Primary One Health domain	Socio-behavioural factors	Research setting	Source of funding
Aggarwal, S., et al. (2021)	Attitudes and awareness about antimicrobials usage and resistance in Delhi, India.	India	Empirical	Quantitative	India	Human health	Age, class, geography, education	University, pharmacies	None stated
Bamidele, O., et al. (2022)	Antimicrobial usage in smallholder poultry production in Nigeria.	Nigeria	Empirical	Quantitative	Nigeria	Animal health	Age, class, geography, occupation, education	Farms	CGIAR Research Program for Agriculture for Nutrition and Health (A4NH)
Barasa, V., et al. (2022)	Using intersectionality to identify gendered barriers to health-seeking for febrile illness in agro-pastoralist settings in Tanzania.	UK	Empirical	Mixed methods	Tanzania	Human and animal health	Age, marital status, disability, geography	Community	Biotechnology and Biological Sciences Research Council, Department for International Development, the Economic and Social Research Council, the Medical Research Council, the Natural Environment Research Council and the Defence Science and Technology Laboratory, under the ZELS-AS programme
Boffa, J, et al. (2018)	The role of agency in the implementation of Isoniazid Preventive Therapy (IPT): Lessons from oMakoti in uMgungundlovu District, South Africa.	Canada	Empirical	Mixed methods	South Africa	Human health	Geography	Community	International Development Research
Chikovore, J. (2017)	TB and HIV stigma compounded by threatened masculinity: Implications for TB health-care seeking in Malawi	South Africa	Empirical	Qualitative	Malawi	Human health	Age, occupation, class	Healthcare facility	Wellcome Trust
Chukwu, E. (2020)	A national survey of public awareness of antimicrobial resistance in Nigeria.	Nigeria	Empirical	Quantitative	Nigeria	Human health	Geography, education	Household	No funding received
Gemeda et al. (2020)	Antimicrobial use in extensive smallholder livestock farming systems in Ethiopia: Knowledge, attitudes, and practices of livestock keepers.	Ethiopia	Empirical	Quantitative, cross-sectional study	Ethiopia	Animal health	Age, sex, education, income source	Agro-ecological zones and mixed crop-livestock production system	The Animal Health Flagship of the CGIAR Research Program on Livestock, CGIAR Research Program on Agriculture for Nutrition and Health, Ethiopia
Govender (2017)	The role of gender in patient-provider trust for TB treatment.	South Africa	Empirical	Qualitative	South Africa	Human health	Geography	Healthcare facility	Department of Science and Technology and NRF South Africa
Islam et al. (2022)	Knowledge, attitudes and practices regarding antimicrobial usage, spread and resistance emergence in commercial poultry farms of Rajshahi district in Bangladesh	Bangladesh	Empirical	Quantitative, cross-sectional study	Bangladesh	Animal health	Age, sex, education, marital status, family income, source of income	Commercial poultry farm	Ministry of Science and Technology, People’s Republic of Bangladesh
Jones et al. (2022)	Gender and antimicrobial resistance: What can we learn from applying a gendered lens to data analysis using a participatory arts case study?	UK	Empirical	Qualitative	Nepal	Human and animal health	Marital status	Community	Global Challenges Research Fund
Kalam et al. (2022)	Knowledge, attitudes, and common practices of livestock and poultry veterinary practitioners regarding the AMU and AMR in Bangladesh.	Bangladesh	Empirical	Quantitative, cross-sectional study	Bangladesh	Animal health	Age, sex, education, years of work, current workplace type, training on AMU,	Livestock and poultry veterinarians	Bangladesh Bureau of Education Information and Statistics
Manderson, L. (2020)	Prescribing, care and resistance: Antibiotic use in urban South Africa.	South Africa	Empirical	Qualitative	South Africa	Human health	Class	Healthcare facility	UK Economic and Social Research Council (ESRC)
Manyau, S. et al. (2022)	Antibiotics and the biopolitics of sex work in Zimbabwe.	UK	Empirical	Qualitative	Zimbabwe	Human health	Class, occupation	Community	UK Aid through the Foreign, Commonwealth & Development Office, United Kingdom Government
Nasir, A. et al. (2019)	Knowledge of antibiotic use, misuse and antibiotic resistance in the slum community in Karachi.		Empirical	Quantitative	Pakistan	Human Health	Class, marital status, geography	Community	None stated
Palumbo, P.J. et al. (2021)	Uptake of antiretroviral treatment and viral suppression among men who have sex with men and transgender women in sub-Saharan Africa in an observational cohort study: HPTN 075.		Empirical	Qualitative	Sub-Saharan Africa	Human Health	Sexuality	Community/network	Division of AIDS Research of the U.S. National Institutes of Mental Health (NIMH)and the Division of AIDS of the U.S. National Institute of Allergy and Infectious Diseases (NIAID), NIMH
Pham-Duc et al. (2019).	Knowledge, attitudes and practices of livestock and aquaculture producers regarding antimicrobial use and resistance in Vietnam.	Vietnam	Empirical	Quantitative, cross-sectional study	Vietnam	Animal health	Age, sex, education level	Small and medium scale pig, poultry and aquaculture farm	USAID
Pham-Duc, P. (2021)	Exploring gender differences in knowledge and practices related to antibiotic use in Southeast Asia: A scoping review.	Vietnam	Systematic Review	Systematic / literature review	South East Asia region	Human, animal, plant health	Age, class, education	Households, farms, schools	No funding received
Salihu Dadari, H., et al. (2020)	Antibiotics use, knowledge and practices on antibiotic resistance among breastfeeding mothers in Kaduna state (Nigeria).	Spain	Empirical	Quantitative	Nigeria	Human health	Class, education	Healthcare facility	No funding received
Simon, B., et al. (2020)	Prevalence and factors associated with parents self-medicating under-fives with antibiotics in Bagamoyo District Council, Tanzania: A cross-sectional study.	Tanzania	Empirical	Quantitative	Tanzania	Human health	Class	Community (households)	None stated
Strom, G., et al. (2018)	Antimicrobials in small-scale urban pig farming in a lower middle-income country: Arbitrary use and high resistance levels.	Sweden	Empirical	Quantitative	Cambodia	Animal health	Education	Farms	Swedish Research Council
Torres, N., et al. (2019)	Evidence of factors influencing self-medication with antibiotics in low and middle-income countries: a systematic scoping review.	Mozambique	Systematic review	Systematic review	Sub Saharan Africa and SEAR	Human health	Geography, age	Universities, hospitals, primary healthcare centres, pharmacies, and households	Higher Institute for Health Sciences Maputo (ISCISA), Norwegian high education and development (NORHED) and UKZN
Zeru, N., et al. (2020)	Self-medication practice and associated factors among University of Gondar College of Medicine and Health Sciences students: A cross-sectional study.	Ethiopia	Empirical	Quantitative	Ethiopia	Human health	Class, education	University	No funder acknowledgment included

As the following sections will demonstrate, these themes overlap to differentially shape women’s and men’s agency in decision-making and their access to resources, with implications for AMR mitigation. We contextualise the themes in relation to the broader literature on gender and human and animal health.

### Gendered division of roles and responsibilities

The review findings reflect the persistence of deep-seated attitudes and beliefs about women’s and men’s roles in care work, with women primarily responsible for household chores and the health and wellbeing of children, adults requiring significant care, and other family members. Considering the dominance of women’s role in attending to the health needs of others, several studies in the review concerned with AMR and human health centre on women, as caregivers, potentially fuelling the development of AMR. This is clearest in the literature concerned with mothers’ knowledge, attitudes and practices regarding their use of antibiotics they give to their children (Salihu Didari et al., 2020; Simon and Kazaura 2020). Research conducted in Nepal highlights how women’s central role as caregivers positions them as custodians of knowledge about antibiotics and their administration to others (Jones et al. 2022):

[R]espondents of both genders reflect that women (in particular wives/mothers) are the ones who should/do know about the medicines given to children […] Women are seen as the ones in the household that have been taught about how and when to give children antibiotics, and that they are responsible for receiving and acting upon this information (p. 5).

Thus, the literature is both descriptive of social norms, and prescriptive– of what women should know and what they should do. Following this focus of AMR research on women as caregivers, AMR awareness-raising efforts, as these relate to human health, often target women (Jones et al. 2022; Manderson 2020; Simon and Kazaura 2020). Such programmes, while important, risk perpetuating unequal gender norms, and in efforts to address AMR, may alienate men, or treat them as irrelevant. Moreover, a limitation in this literature is the narrow focus on women as parents of young children. Family structures in LIMCs are diverse (Morison et al. 2019). Women care not only for children but also for elderly family members, partners who are ill and, in many instances, their extended family. Moreover, in multigenerational households, caregiving often falls on younger wives and other women (Boffa et al. 2018). In the context of care, women must manage a range of infections– urinary tract infections, infected bites, lesions, cuts and rashes, and seasonal and other respiratory infections. In some settings, who can undertake caregiving is influenced by employment opportunities and the rural-urban migration of working-age men and women. In SSA, until recent years, the HIV epidemic resulted in older women often assuming the role of primary caregivers, and still, rural women typically care for and receive care from granddaughters and great-granddaughters (Small et al. 2019). The studies under review, however, primarily focus on a nuclear family pattern of care and AMU in LMICs, suggesting adopting a Western family structure of biological mothers and their children, and to some extent rural-urban migration, where this pattern is disrupted. Findings may, therefore, not hold relevance across the diversity of family and household structures in LMICs (Morison et al. 2019).

Women’s domestic role extends to caring for small-scale livestock and subsistence gardens. Their daily activities include feeding, cleaning, watering, milking, and sometimes herding animals, and collecting manure to enrich domestic gardens. Since women spend more time with livestock, they observe animals for signs of disease and treat sick individuals, administering traditional and modern medicines (Miller 2011a, b). Men have more mobility and are responsible for breeding and fodder production; hence, men are more likely to interact with extension agents and animal health specialists (Miller 2011b). Livestock for sale at the market continues this gendered division of labour, with men controlling marketing and finances. Community narratives in Nepal about AMR in farming practices echo gendered perceptions of women exclusively considered in relation to animal-rearing chores, with discussions about farming practices centred on men (Jones et al. 2022). Miller (2011a) notes that “institutions delivering animal healthcare or training have a male bias, and direct resources to the male head of household” (p. 18). Women’s role in livestock-keeping of large animals, particularly, is labelled as ‘helpers’ instead of co-owners (Galiè et al. 2022; p. 3). This gendered division of livestock management results in women being overlooked in recruitment for animal health AMR projects (Acosta et al. 2022; Kruijssen et al. 2018). Extension services and animal vaccination campaigns often target men, either because women are not recognised as farmers or because the timing and location of animal healthcare campaigns make it difficult for women juggling multiple responsibilities to make the trip (Acosta et al. 2022; Kruijssen et al. 2018).

In aquaculture, women’s contributions are similarly undervalued and seen as part of their domestic duties (Aung et al. 2021; FAO 2017; Treviño and Murillo-Sandoval 2021; Wulandari 2020). In their research about aquaculture in South East Asia, Sari and colleagues (2017) note that even when women have ownership of ponds, they face overt social criticism (aquaculture is considered a masculine activity), and control of the use of productive assets is still male-dominated. Consequently, AMR mitigation training resources are directed to the male operators or heads of households (Ström et al. 2018). These findings indicate a lack of acknowledgement of women’s presence and contributions to decision-making and a lack of attention to their possible role in AMR prevention in agriculture, detracting from the impact and sustainability of AMR mitigation interventions.

Several of the studies under review comment on overlapping religious and gender norms about the reproductive and domestic roles of women and the household as the site of women’s work, and how this impacts women’s access to AMR mitigation resources. In research conducted in SEA, some women working in household aquaculture operations cannot access public awareness campaigns, information services, veterinary services or community networks without a male escort (Pham-Duc et al. 2019; Wulandari 2020). Research in Kenya and Uganda describe similar local norms about women livestock keepers: women require their husbands’ permission to travel, impacting their ability to freely travel to AMR information or training activities (Bikaako et al. 2022; Kyotos et al. 2022). Financial barriers to vaccines, limited access to and sometimes exclusion from extension services and resources, and marketing mechanisms hinder women from gaining knowledge about prevention practices and the awareness of how to demand vaccines to prevent infection toward themselves and their animals.

### Gender power relations in decision-making and access to resources

As illustrated above in the first theme, traditional gender norms that assign caregiving tasks to women, such as seeing to the health needs of children and other relatives, put women at the centre of household antibiotic use. Yet, unequal gender norms mean that women often need permission from their husbands to attend a healthcare facility, pay for transport or purchase medication. Men are widely and commonly considered the households’ primary decision-makers, including whether and when household members can seek healthcare (UNDP 2021). Barasa and Virhia (2022) explain:

Many women interviewed disclosed that where treatment involved financial costs, men made the decision. Women, however, generally sought treatment earlier and made decisions where treatment was free; for example, obtaining herbal remedies from nearby bushes or borrowing medicine from family and friends, and where possible, obtaining cheaper alternative drugs from informal sellers rather than from clinics or pharmacies (p. 11).

In some sociocultural contexts, women might also need permission to seek treatment from extended family and, in polygamous households, from older/first wives. Barasa and Virhia (2022) explain how a participant, Neema (pseudonym), a woman in a polygamous marriage wishing to seek diagnosis and treatment, had her decision overridden by her husband and his first wife:

[Neema] had the money necessary to attend the local clinic to be examined by a clinician but could not simply go without first consulting and getting permission from her extended family. Her husband wanted to consult his first wife in advance of Neema making the journey, because this first wife was older and was perceived to be more experienced in interpreting illness symptoms. Moreover, in this case, the husband thought that Neema’s condition was improving and thus that she did not need to go to the doctor. Although he perceived the illness to be ‘severe’ fever, he did not believe it to be a ‘hospital disease’ worthy of spending money on […]. The first wife shared the husband’s view and so Neema’s decision to visit the doctor was curtailed (p. 13).

While decision-making is mainly discussed in the studies under review in relation to human healthcare, a similar finding emerges concerning women’s roles in aqua- and agriculture. For instance, in research conducted in Indonesia, men are the primary decision-makers in small-scale aquaculture, even when women are lead operators. If differences of opinion arise, women follow their husbands’ preferences (Sari et al. 2017). Participants in a study conducted in Nepal identified gender-based violence (GBV) as a potential consequence for women who “do not fully comply with their husbands’ wishes” (Jones et al. 2022; p. 10). Consequently, if women do not have their male partner’s support in their treatment-seeking, they are left with fewer options to adhere to guidelines for appropriate antibiotic use, meant to curb the development of AMR. Women’s agency in healthcare decision-making is especially constrained by men’s control of household financial resources, impacting women’s ability to travel to health facilities, pay user fees and purchase medication (Barasa and Virhia 2022; Jones et al. 2022).

For especially poor women and women living in rural areas, these risks are even more pronounced, pointing to the importance of attending to intersections between gender and poverty in AMR research. For example, a study conducted in Tanzania found that 47% of mothers reported giving their ill children unprescribed antibiotics, often purchased from informal pharmacies in their community, because they did not have money to travel to healthcare facilities (Simon and Kazaura 2020). Underscoring intersections between gender, class and area of residence (notably rural areas), the authors note that the use of unprescribed antibiotics by mothers is higher for those living further from health facilities. Govender (2017) also cites distance to healthcare facilities as a barrier to women’s treatment adherence. Manderson (2020) notes the relevance of intersections between gender and area of residence in peri-urban areas, too:

Women without the cash to pay for a taxi minibus or bus might walk for half an hour to an hour […] Women reported borrowing from others to cover the indirect costs of clinic attendance, such as transport and possible charges for medication (p. 4).

A Zimbabwean study about sex workers’ AMU brings the tensions between gendered decision-making power, women’s economic vulnerability, and AMR risk into stark focus (Manyau et al. 2022). While unprotected sex with clients exposes sex workers to sexually transmitted infections (STIs) and, consequently, higher AMU and risk of resistant infections, their economic vulnerability limits their agency. Manyau and colleagues (2022) describe, “[i]n theory, sex workers could choose to engage only in protected sex. However, this was an ‘empty choice’ for most, due to the significant difference in price between protected and unprotected sex” (p. 264). A sex worker explains, “Why not get better money to pay my bills and feed my children? Only those with less responsibilities have the luxury to play it safe” (Manyau et al. 2022; p. 264). Jones and colleagues (2022), in their research on gender norms in household decision-making, note a similar tension inherent in holding women responsible for appropriate AMU in managing their health and that of others in their care, without engaging men as primary decision-makers about financial resources needed to access healthcare.

### Interactions between gender norms, health-seeking behaviour and AMU

Several studies focused on gender norms regarding appropriate behaviour by women and men and the implications for women’s and men’s health-seeking behaviour and, consequently, AMU. Gendered socio-cultural norms that emphasise women’s demonstration of obedience to and respect for men may influence women’s agency in healthcare decision-making. In their research about adherence to TB preventive therapy, Boffa and colleagues (2018) describe how social sanctions against women asking “too many questions” or “knowing too much” may limit the extent to which they take control of decisions to initiate or continue treatment. Instead, they defer to healthcare providers’ expertise. This might translate into higher treatment adherence, but it may also result in a lack of transparency when experiencing challenges with side effects or drug interactions for fear of appearing disrespectful in healthcare-provider interactions, leading to interruptions in or discontinuation of treatment (Boffa et al. 2018).

Similarly, several studies describe how gender norms about masculinity contribute to men’s AMR risk. Gendered expectations of men to be strong, healthy and resilient can contribute to their avoiding or delaying treatment-seeking, especially when they perceive health-seeking as indicative of weakness or vulnerability (Barasa and Virhia 2022; Chikovore et al. 2017; Govender 2017; Jones et al. 2022). When men do seek treatment, they might opt for obtaining antibiotics without a prescription, leaving them without education on appropriate use. The authors of a community-based study conducted in Nepal describe how social norms about masculinity may contribute to men placing pressure on health staff to prescribe antibiotics, even if not clinically indicated, since ‘strong’ antibiotics are associated with quicker recovery (Jones et al. 2022).

When treatment regimens are perceived as a direct threat to behaviours considered masculine, men may deprioritise health-seeking behaviour, opt to access non-prescribed antibiotics, and/or terminate their treatment early (Chikovore et al. 2017; Govender 2017). For example, a study conducted in Malawi reports how tuberculosis (TB) treatment is perceived as requiring lifestyle changes that disrupt behaviours strongly associated with masculinity, such as requirements to abstain from alcohol consumption and smoking (Chikovore et al. 2017). Consequently, normative notions of masculinity may impact men’s willingness to remain in TB treatment and care, ultimately contributing to AMR (Chikovore et al. 2017). On the other hand, the authors also describe how some men in their study resisted normative expectations of invulnerability and actively engaged their status as ill, which allowed them to pursue healthier behaviours such as avoiding alcohol use and smoking, adhering to treatment, and seeking psychosocial support. Overall, however, there is little research in the dataset speaking to challenges to harmful gender norms, practices or power relations that fuel AMR risk.

Significantly, the review findings do not reflect the same attention to the influence of gender norms in AMU decisions related to animal agriculture compared to the literature focused on human health.

## Discussion and recommendations

The findings provide insight into several critical evidence gaps and priorities, and underscore the need for further research, deepening the evidence about the relationship between gender-based and other equity considerations and AMR. Sustainable and gender-equitable AMR containment and mitigation efforts require a nuanced understanding of how gendered power relations, roles and responsibilities shape AMU decision-making, risks and vulnerabilities. Considering the impact of local context on gender norms, further research must reflect diversity in geographic and socio-cultural settings across LMICs. Related to this, there is a need for more research about men’s role in decision-making about their partners’ and other household members’ health-seeking behaviour, to better understand how men can be engaged in dismantling rigid gender norms that constrain women’s agency. Qualitative research with men as participants will be valuable to this end. Except for Chikovore et al. (2017), there is a lack of research exploring how harmful gender norms, practices or power relations that contribute to AMR risk can be challenged and transformed.

The findings highlight women’s role in tending to the health of children and other family members and consequently, women’s prominent role in household and community AMR stewardship, relative to men. Targeting women in awareness and education campaigns is therefore a clear opportunity for AMR mitigation efforts in human health. It is important, however, for AMR mitigation to avoid focusing on women in isolation from the settings in which they are making choices about AMU, and thereby inadvertently perpetuating narratives that blame women for adverse AMU outcomes in their households and communities. Public health policies, programmes and interventions that situate women as AMR custodians can mitigate this by embedding a nuanced understanding of the conditions in which women use antibiotics– in LMICs, this is often in precarious living environments, with limited income generating activities and constrained decision-making. Considering the diversity of family and household structures globally but particularly in LMICs, such research should include caregiving in relation to other family members, including extended family, and across diverse family structures, e.g., multigenerational and polygamous families.

In our discussion of animal caretaking, we drew attention to the frequent disregard of women’s roles and their exclusion from extension activities addressing animal health and disease prevention. This is not a new point; the systematic exclusion of women from training, outreach and credit has been raised recurrently over the past 40 or more years (Bettles 1980; Lewis 1981; Berger et al. 1984). Further, the broader literature on women and agriculture, women’s decision-making, and gendered divisions of household labour and care work all date from this period. In part, the scoping review has highlighted the quarantining of literature, with little evidence of the translation of the work of rural sociologists, anthropologists and others to the field of One Health and environmental health, even less concerning the social factors that contribute to AMR.

There are important gaps in the literature related to AMR, gender and public healthcare settings. Research conducted in HICs indicates prescriber bias, where women are more likely to be prescribed antibiotics than men (Schröder et al. 2016). The findings show that literature produced in LMICs shows scant attention to this area of investigation, except for Manderson (2020) describing the role of class-based assumptions in AMU prescribing. The study highlights the impact of healthcare providers’ assessment of the socio-economic circumstances of women on their antibiotic prescribing practices, where the financial burden of returning to a clinic if their condition does not improve can influence the decision to provide an antibiotic prescription during the first visit, even if not clinically indicated (Manderson 2020). Future research can explore provider AMU practices and beliefs in LMICs. Further, research in HIC settings have explored how women’s overrepresentation in care work occupations can increase their AMR risk. This includes “frontline healthcare workers,” workers in related fields such as cleaners, and in residential care, who are exposed occupationally to drug-resistant organisms (DRO). While women’s vulnerability as frontline healthcare workers has been explored in the context of the COVID-19 pandemic (e.g., Small et al. 2020), similar LMIC studies focused on AMR were not identified in the scoping review.

Finally, the findings show limited attention to intersectionality in research about AMR and gender. It will be important for future research to engage other socio-cultural and structural factors overlapping with gender, in addition to class and geographic location, to better direct the development of programmes, policies and interventions that are responsive to how AMR risk and vulnerability manifest in resource-constrained settings. Further to this, the review indicates that the current evidence base overwhelmingly treats gender as binary, despite evidence that sexual and gender diversity are important social determinants of health (Zeeman et al. 2019). Integrating an intersectional lens can deepen understanding of overlapping of socio-cultural and structural inequalities relevant to the reciprocal relationship between gender inequality and AMR.

### Limitations

While every effort was made to identify relevant literature, the review has limitations, mainly by excluding books and book chapters. Further, the authors conducted searches on aggregator databases only, the topic of gender and equity in AMR literature may also be prevalent in more discipline specific databases (e.g., natural sciences, veterinary sciences). Finally, while the review findings show some diversity in research country context, author location, research setting and source of funding (see Table 1), the small size of the dataset does not allow for a more nuanced analysis of these trends in gender and AMR knowledge production.

## Conclusion

The research highlights that gender norms and power relations matter concerning access to health care, responsibility for other humans and other animals, and as a target of health education and agricultural outreach. At the same time, the range of issues captured in this review emphasises that people of any gender may be vulnerable to AMR, and resist or be denied health care and treatment support, because of stigma or normalising beliefs. All people are made vulnerable in this context. Research that fails to account for gender and its intersections with other lines of disadvantage, such as race, class and ability, risks being irrelevant with little impact on the continued and dangerous spread of AMR.
